# Adapting HIV services for pregnant and breastfeeding women, infants, children, adolescents and families in resource‐constrained settings during the COVID‐19 pandemic

**DOI:** 10.1002/jia2.25622

**Published:** 2020-09-01

**Authors:** Alexandra C Vrazo, Rachel Golin, Nimasha B Fernando, Wm P Killam, Sheena Sharifi, B Ryan Phelps, Megan M Gleason, Hilary T Wolf, George K Siberry, Meena Srivastava

**Affiliations:** ^1^ Office of HIV/AIDS United States Agency for International Development Washington DC USA; ^2^ US Centers for Disease Control and Prevention Atlanta GA USA; ^3^ Office of the U.S. Global AIDS Coordinator and Health Diplomacy Washington DC USA

**Keywords:** adolescents, children, COVID‐19, family‐centred |, HIV, maternal

## Abstract

**Introduction:**

The COVID‐19 pandemic has impacted global health service delivery, including provision of HIV services. Countries with high HIV burden are balancing the need to minimize interactions with health facilities to reduce the risk of COVID‐19 transmission, while delivering uninterrupted essential HIV prevention, testing and treatment services. Many of these adaptations in resource‐constrained settings have not adequately accounted for the needs of pregnant and breastfeeding women, infants, children and adolescents. We propose whole‐family, tailored programme adaptations along the HIV clinical continuum to protect the programmatic gains made in services.

**Discussion:**

Essential HIV case‐finding services for pregnant and breastfeeding women and children should be maintained and include maternal testing, diagnostic testing for infants exposed to HIV, index testing for children whose biological parents or siblings are living with HIV, as well as for children/adolescents presenting with symptoms concerning for HIV and comorbidities. HIV self‐testing for children two years of age and older should be supported with caregiver and provider education. Adaptations include bundling services in the same visit and providing testing outside of facilities to the extent possible to reduce exposure risk to COVID‐19. Virtual platforms can be used to identify vulnerable children at risk of HIV infection, abuse, harm or violence, and link them to necessary clinical and psychosocial support services. HIV treatment service adaptations for families should focus on family based differentiated service delivery models, including community‐based ART initiation and multi‐month ART dispensing. Viral load monitoring should not be a barrier to transitioning children and adolescents experiencing treatment failure to more effective ART regimens, and viral load monitoring for pregnant and breastfeeding women and children should be prioritized and bundled with other essential services.

**Conclusions:**

Protecting pregnant and breastfeeding women, infants, children and adolescents from acquiring SARS‐CoV‐2 while sustaining essential HIV services is an immense global health challenge. Tailored, family friendly programme adaptations for case‐finding, ART delivery and viral load monitoring for these populations have the potential to limit SARS‐CoV‐2 transmission while ensuring the continuity of life‐saving HIV case identification and treatment efforts.

## 1 INTRODUCTION

As the impact of the COVID‐19 pandemic unfolds, the high person‐to‐person, nosocomial and community transmission rates of severe acute respiratory syndrome coronavirus 2 (SARS‐CoV‐2) [1] – including from asymptomatic carriers – are placing an immense burden on healthcare systems and vulnerable populations who may be increasingly deterred from accessing care [2]. In resource‐constrained settings with HIV disease burden, ensuring continuity of safe case identification and life‐saving, life‐long antiretroviral therapy (ART) remains a global health priority [3]. Little data are available on the impact of the COVID‐19 epidemic on established healthcare systems designed to respond to the HIV epidemic; therefore, global health partners and Ministries of Health are working to develop and adapt guidance for healthcare systems, including supply chain, healthcare workforces and communities to reduce risk of exposure to COVID‐19 while avoiding interruptions to essential HIV services [4, 5, 6].

Data are currently limited on whether people living with HIV (PLHIV) are more likely to acquire SARS‐CoV‐2, or if COVID‐19 disease progression is different among PLHIV [7, 8]. A recent analysis from South Africa reveals that PLHIV, especially those with comorbidities, have elevated risk of poor COVID‐19 outcomes, irrespective of viral suppression [9]. However, information is scarce on the potential impact of HIV/SARS‐CoV‐2 co‐infection on vulnerable populations including pregnant and breastfeeding women (PBFW), HIV‐exposed infants (HEI), children/adolescents living with HIV (C/ALHIV) and orphans and vulnerable children (OVC).

Although some COVID‐19 health service adaptations address the needs of PBFW, HEI, C/ALHIV and OVC, many do not. Aligning with updated PEPFAR guidance, we propose programme adaptations along the clinical continuum to protect the gains made in combating the global HIV epidemic among these high‐risk populations during the COVID‐19 response (Table 1). These adaptations should be supported by tailored community messaging to reassure clients who may otherwise be fearful of COVID‐19 infection risk and may not access routine services. Programme adaptations can also be utilized during times of high community transmission of COVID‐19, especially when mitigation measures and travel restrictions are in place, and access to health facilities is reduced.

**Table 1:** Summary of essential HIV services, programme adaptations and country‐level actions/solutions for pregnant and breastfeeding women, infants, children and adolescents during COVID‐19

Service	Delivery mode		Primary recipients of service (implement in accordance with national guidelines)			
	In‐Person (F = Facility, C = Community)	Virtual	PBFW	Infants	Children	Adolescents
HIV Risk Screening	✓ (F,C)	✓	✓ HIV‐negative women at prenatal or postpartum visit	All HEI should receive EID services	✓	✓
	Adaptation/ Intervention Examples	Kenya Virtual risk screening for AGYW as part of the DREAMS Virtual Safe Space layered package of services [A1]				
HTS (blood‐based)	✓ (F)		✓	✓	✓	✓
	Adaptation/ Intervention Examples	Kenya Prioritizing HTS for ANC, EID, partner/family/index testing and other sub‐populations specified in guidance [34] Zimbabwe Development of information, education and communication materials tailored for C/AYLHIV (0‐24 y/o), caregivers, health service providers, social workers, faith/religious leaders; aim to make information about COVID‐19, HTS, PMTCT, mental health, SRH and other topics accessible during COVID‐19 through radio sessions, comic scripts, animations and other developmentally appropriate materials [A2]				
HIVST (oral screening)	✓ (F,C)		✓		✓ Children ≥ 2 years of age	✓
	Adaptation/ Intervention Examples	Kenya AGYW enrolled in DREAMS Virtual and Mobile Safe Spaces are offered the option to HIVST at home using an OraQuick kit delivered to their home by a volunteer; volunteer or DREAMS mentor accompanies client to HF for confirmatory diagnosis/linkage to treatment [A1] Zambia Increasing availability of HIVST at adolescent‐friendly facility‐based testing sites to increase adolescent partner index testing [B1]				
EID Services	✓ (F,C)			✓	✓ (as applicable to ascertain final PMTCT outcome)	
	Adaptation/ Intervention Examples	Burundi DBS sample taken from HEI; results are returned during the next ART drop off [B3] DRC, Tanzania, Zimbabwe Community‐based EID testing or sample collection for EID [B2] [C1] [C2] Kenya, Zimbabwe Integrating EID testing with immunization appointments/co‐delivery of EID [C3] [C2] Zambia m‐PIMA cartridges readily available for birth testing; RAL granules for first 28d of life provided for HIV + neonates [B4]				
Post‐ART initiation clinical follow‐up	✓ (F,C)	✓	✓	✓	✓	✓
	Adaptation/ Intervention Examples	Uganda Linkage facilitators, youth peers, HF staff, parish‐based volunteers and others coordinate to decongest ART clinics by line listing clients for drug refills a week before their appointment date and distributing ART at community distribution points [A3]				
MMD (ART initiation and refills)	✓ (F,C)		✓		✓ (>2 years of age)	✓
a) PBFW	Adaptation/ Intervention Examples	Eswatini Aligning ART refills for PBFW with scheduled ANC and post‐natal visits; if client is stable on oral contraceptives, align contraceptive refill and ART refills [35] Malawi, Mozambique, Tanzania, Zimbabwe 3‐6‐month MMD for PBFW [D1] [C1] [C2] South Africa Permitting 6MMD extension of certain prescriptions including TLD, all second line ARV, and TEE for women of childbearing potential for patients with a contactable cell phone number; use of verbal counselling during medication pick‐up and SMS to inform patients of extensions; WhatsApp, toll‐free call centre, and other methods used for communication; created a new functionality on SyNCH to identity patients with prescriptions eligible for extension [36]				
b) C/ALHIV	Adaptation/ Intervention Examples	Cameroon, DRC, Kenya, Nigeria, Uganda, Zimbabwe CRS’ OVC programmes coordinating with clinical partners to operationalize MMD [B5] Eswatini, Malawi, Tanzania, Zambia, Zimbabwe 3‐to 6‐month MMD for C/ALHIV [35] [D1] [C1] [C2] South Africa Ages 5‐18y/o eligible for Repeat Prescription Collection if on ART for over 6m, no regimen/dosage change in the last 3m, VL less than 6m old. and VL less than 50copies/mL; caregivers should be counselled on disclosure [37]				
Viral load monitoring	✓ (F,C)		✓	✓	✓	✓
	Adaptation/ Intervention Examples	DRC Coordinate VL sample collection with ART pick‐up or other essential clinic appointment (e.g. childhood immunizations) for children < 20kg and PBFW [B2] Nigeria Pregnant women are among the PLHIV prioritized for VL testing; client engaged virtually to agree on time and place for sample collection; if VL collection in the HF, then appointments are staggered. This strategy has resulted in VL testing in the community and weekly increases in VL testing since lockdown started [A4]. Uganda Line list, map, and cluster clients due for VL testing, focus on C/ALHIV < 14 y/o living within 25km radius of the facility; Locum counsellors pick up samples (DBS preferred over serum); motorcycle transport; para‐social workers, linkage facilitators, and village health teams used to reach mapped households [B6]				
Return of viral load results	✓ (F,C)	✓	✓	✓	✓	✓
	Adaptation/ Intervention Examples	Lesotho Prioritized infants and PBFW for VL monitoring; results fast‐tracked through an e‐lab and strong collaborations between clinicians and laboratory staff [A5]				
Provision of TPT and CTX Prophylaxis	✓ (F,C)		✓	✓	✓	✓
	Adaptation/ Intervention Examples	Eswatini CLHIV on ART eligible for 3MMD and taking prophylactic medications including TPT, CTX, and fluconazole should also receive longer refills of these medications [35] Ethiopia HEI to receive 3MMD of combined HIV infant prophylaxis and CTX; breastfeeding mothers also to receive 3MMD; one person should pick up drugs for the family; telephonic counselling by healthcare workers [D1]				
Adherence Support	✓ (F,C)	✓	✓	✓	✓	✓
	Adaptation/ Intervention Examples	Kenya Mentor Mothers call clients before adherence support clinic visits; if the client is not reached or declines to present for ART pick‐up, a treatment buddy or closest community volunteer is asked to contact the client [C3] South Africa Virtual/phone‐based adherence monitoring using a USAID approved service tool to structure and standardize phone‐based adherence monitoring among CLHIV [B7] Uganda Increased collaboration with HFs to transport health officers to home visits, collect in‐home blood samples for VL testing and monitoring, adherence counselling support, follow‐up OVC who missed appointments or require ART refills or who are not virally suppressed [B8]				
Psychosocial support	✓ (F,C)	✓	✓	✓	✓	✓
	Adaptation/ Intervention Examples	Lesotho Psychologists offer support to mothers of C/ALHIV who may be receiving MMD; provide information on drug supply and reminders for next appointment [B9] Kenya DREAMS Mobile Safe Spaces allow AGYW to meet for a socially distanced group session and receive a layered package of services including referrals/appointments for HTS, PrEP, GBV and STI screening [A1]				
Referral to OVC, GBV, and community support	✓ (F,C)	✓	✓	✓	✓	✓
	Adaptation/ Intervention Examples	South Africa Coordinating with partners to enroll and serve OVC beneficiaries during delivery of food parcels to homes; HF refers new CLHIV to OVC by phone; adapted messaging for delivery via telephone, WhatsApp, online, or SMS; providing printed educational materials to OVC households without smartphones or data [B7] Tanzania Offering virtual age‐appropriate first‐line support to all clients that disclose intimate partner violence during index testing; SMS system to virtually monitor post‐GBV care service delivery; target beneficiaries include PLHIV, women of reproductive age, and adolescents [A6] Zambia Using Childline/Lifeline to deliver COVID‐19 messaging and psychosocial support within the GBV‐focused programme; supporting GBV survivors in shelters with COVID‐19 prevention items such as hand sanitizer; target beneficiaries include children 9‐14 y/o, AGYW, and adolescent boys and young men [A7]				

AGYW, adolescent girls and young women; ANC, antenatal care; ART, antiretroviral therapy; C/AYLHIV, children, adolescents, and youth living with HIV; CLHIV, child living with HIV; CRS, Catholic Relief Services; CTX, cotrimoxazole; DBS, dried blood spot; DRC, Democratic Republic of the Congo; DREAMS, Determined, Resilient, Empowered, AIDS‐free, Mentored, and Safe Partnership; EID, early infant diagnosis; GBV, gender‐based violence; HEI, HIV‐exposed infants; HF, health facility; HIVST, HIV self‐testing; HTS, HIV testing services; MMD, multi‐month dispensation of ART; OVC, orphans and vulnerable children; PBFW, pregnant and breastfeeding women; PMTCT, prevention of mother‐to‐child transmission of HIV; PrEP, pre‐exposure prophylaxis; RAL, raltegravir; SMS, short message service; SRH, sexual and reproductive health; STI, sexually transmitted infection; TEE, tenofovir/emtricitabine/efavirenz; TLD, tenofovir/lamivudine/dolutegravir; TPT, TB preventive therapy; USAID, United States Agency for International Development; VL, viral load.

References Codes

[A#] Presentations at USAID’s Partner Operational Solutions in Response to COVID‐19 Meeting, [A1] (PATH, Afya Ziwani: “ART and PrEP in the time of COVID‐19: Leveraging messaging/video platforms and community‐based delivery systems,” April 28, 2020;), [A2] (Zvandiri Africaid, Zimbabwe: “Improving COVID‐19 Awareness, Prevention Actions and Support Among 0‐24 Year Olds in Zimbabwe,” June 10, 2020), [A3] (University Research Co., LLC and IntraHealth International, Uganda: “Human Resources Solutions Being Implemented at Health Facilities in the Context of COVID‐19: Regional Health Integration to Enhance Services‐East Central, East and North‐Acholi (RHITES‐EC, RHITES‐E, RHITES‐N. ACHOLI),” April 24, 2020), [A4] (SIDHAS‐FHI, Nigeria: “Scaling up Viral Load Sample Collection amid the COVID‐19 Lockdown,” April 30, 2020), [A5] (EGPAF, Lesotho, “Lesotho’s Strategies to Improve Pediatric Viral Suppression Rates: Overcoming the Impact of COVID‐19,” April 30, 2020), [A6] (EngenderHealth & EGPAF, Tanzania: “USAID Boresha Afya Northern Central Zone GBV Focused Solution in Response to COVID‐19 Pandemic,” May 26th, 2020), [A7] (Zambia Center for Communication Programs, Kwatu, Zambia: “USAID Stop GBV Project,” May 26, 2020), [A8] (FHI 360 DREAMS, Zimbabwe: “FHI 360 DREAMS SRH Services Referral Network SRN,” May 26, 2020), [B#] Partner Operational Solutions in Response to COVID‐19 (USAID, written personal communications with Implementing Partners), [B1] (FASTER Zambia, personal communication), [B2] (IHAP‐HK DRC, personal communication), [B3] (PSI Burundi, personal communication), [B4] (CRS/FASTER Zambia, personal communication), [B5] (CRS, personal communication), [B6] (RHITES‐ACHOLI Uganda, personal communication), [B7] (Capacity Development & Support Project South Africa, personal communication), [B8] (BOCY Uganda, personal communication), [B9] (EGPAF Lesotho, personal communication), [C#] Presentations at the Centers for Disease Control, Maternal Child Health, Learning Collaborative meeting, May 19, 2020), [C1] (CDC Tanzania), [C2] (CDC Zimbabwe), [C3] (CDC Kenya), [D#] Personal Communications, [D1] (P Preko, CQUIN, ICAP).

## 2 Discussion: Programme adaptations for maintaining HIV services for specific populations during the COVID‐19 response

### 2.1 Adaptations for case‐finding for undiagnosed PBFW with HIV and their infants

Given the high rates of maternal and infant mortality in resource‐constrained settings with high HIV prevalence, HIV testing in antenatal and maternal child health services for PBFW and their infants should continue during COVID‐19. The adaptation for these HIV services, including maternal testing, retesting, linkage to HIV prevention (e.g. pre‐exposure prophylaxis) [10] for HIV‐negative at risk women [11], and early infant diagnostic testing and prophylaxis, can be provided in a one‐stop‐shop model during routine prenatal, postnatal and child wellness visits. For facility‐based HIV testing in the context of maternal/child health, the adaptation of moving services external to the facility (e.g. wellness tents) may benefit PBFW and their infants while maintaining privacy and confidentiality.

An additional adaptation to maximize access to testing for PBFW is HIV self‐testing kit distribution in the community, provided alignment with national guidelines, the risk screen is negative for intimate partner violence, and the supply chain is intact [12]. If virtual follow‐up is unsuccessful following distribution of HIV self‐tests to PBFW, facility or home‐based visits may be required, and all COVID‐19 precautions should be followed [6]. For clients with initial reactive tests, prioritization should be given to completing the diagnostic algorithm and same‐day linkage to ART. Confirmation of reactive test results and active linkages will be challenging given restrictions on in‐person interactions, thus additional resources will be needed to expand virtual, telephonic, SMS or online follow‐up [6]. Importantly, virtual follow‐up approaches will only be successful if updated and accurate patient contact information is available; this information as well as alternative means of contact should be collected and confirmed during clinical, home‐based or virtual encounters.

For infant testing, as a result of precautions taken during COVID‐19, mentor mothers and facility‐based providers may need to closely support postpartum mothers after birth via SMS and phone consults to instruct on dosages, appointment scheduling and location for sample collection for early infant diagnosis (EID). In Zimbabwe, birth testing for HEI is now being offered at sites with point‐of‐care testing, since mothers and infants may not be able to return for the six‐week EID test given the reduced mobility due to COVID‐19 (CDC Zimbabwe, internal communication). Other countries utilize community‐based EID sample collection and co‐delivery of EID and routine immunization through facility and community‐based platforms; these interventions reduce over‐crowding in health facilities and exposure to COVID‐19 (CDC Tanzania and CDC Zimbabwe, internal communications).

### 2.2 Adaptations for case‐finding for undiagnosed C/ALHIV

During the COVID‐19 pandemic, facility‐based HIV testing services (HTS) remain an important priority, especially for children and adolescents, including those presenting or admitted to health facilities with comorbidities (e.g. tuberculosis, malnutrition, sexually transmitted infections) or other risk factors for HIV. While active case‐finding may temporarily need to be decelerated to ensure the safety and security of providing services during COVID‐19, passive index testing for sexual partners, spouses and biological children of PLHIV newly diagnosed or currently on ART (and child and adolescent siblings of C/ALHIV) who present to the health facility should continue, with immediate linkage to ART for newly identified C/ALHIV [6].

The World Health Organization recently approved healthcare worker‐assisted HIV self‐testing for children two to eleven years of age, which can be employed as an adaptive, decentralized measure if supported by country guidelines [13]. Programmes may also consider, as a temporary adaptation during COVID‐19, dispensing oral‐fluid HIV‐screening kits in the community to parents living with HIV (index clients) to screen biological children for HIV at home, provided that criteria are met (detailed for PBFW above). In Kenya, HIVself‐test kits are being distributed to adolescent girls and young women through community health workers; they are then accompanied for confirmatory diagnosis and linkage to ART if needed (PATH: Afya Ziwani, Kenya, presentation at USAID’s Partner Operational Solutions in Response to COVID‐19 Meeting, April 28, 2020). A written standard operating procedure for home testing, training for healthcare providers, intact supply chain plan, community or facility‐based linkage to treatment and caregiver education to mitigate potential inadvertent social harm should be in place to support this approach [12, 14].

Case identification during COVID‐19 should incorporate virtual approaches within existing OVC and other community case management platforms to allow for continuity of cost‐effective case‐finding while further strengthening collaboration between community and clinical platforms. Risk screening for children and adolescents for HIV, abuse, harm and violence should continue by phone using established risk‐assessment tools, with strong bi‐directional linkage to peer and group support and HIV testing and treatment services if needed [15].

### 2.3 Differentiated service delivery adaptations for PBFW, infants and C/ALHIV

Differentiated service delivery models, including family based multi‐month dispensing (MMD) of ART and community‐based ART services, are potentially impactful programme adaptations for individuals initiating or continuing ART while adhering to national guidelines for COVID‐19. These approaches address the frequency with which clients visit the health facility, and the need for triage, clinic flow and infection control measures [16].

Community‐based ART approaches have demonstrated high retention rates among paediatric and adult clients and are supported for children, adolescents and PBFW [17, 18, 19, 20, 21]. These approaches leverage either the public or private sector for decentralized ART distribution and include fixed community distribution points, mobile outreach ART delivery, home delivery from pharmacies or via mail and adherence clubs [22]. In addition, same‐day ART initiation in the community for PBFW, infants and C/ALHIV is a reasonable alternative to facility‐based initiation coupled with virtual adherence follow‐up [23, 24, 25]. During COVID‐19, community‐based ART services can provide cost savings for clients and facilities by leveraging the health workforce (community health workers, support group members and lay cadres) to provide COVID‐19 prevention education to clients [26], provided that such support does not place health workers at increased risk of COVID‐19 infection and personal protective equipment is available if needed. Where feasible, facility‐based staff can be reallocated to support community‐based ART distribution and other essential activities [6].

Being able to access timely ART refills during the COVID‐19 pandemic is a reported concern among PLHIV and rapid scale‐up of MMD is one strategy to address these concerns [2, 27]. With MMD, families can access longer ART refills from health facilities or through community‐based distribution approaches. Implementation of family‐based MMD within the context of COVID‐19 warrants proactive flexibility with optimized ARV formulations and treatment regimens, as well as bundling with other prescriptions for tuberculosis preventive therapy and cotrimoxazole. If in accordance with national guidelines, providers should ensure that all eligible family members have at least a three‐month supply of ART and when feasible, a six‐month supply [4, 6]. Transition of adolescents and PBFW to tenofovir/lamivudine/dolutegravir should be accelerated with uninterrupted continuation of MMD (if they were already receiving MMD) and virtual follow‐up to assess for side effects and tolerability. For those who miss ART refills, continuing retention support should be adapted through virtual approaches, with home‐based visits only when necessary [6].

Even prior to COVID‐19, family‐based MMD has been recommended for PBFW, yet implementation has been limited [17, 25, 28]. As part of the early response to COVID‐19, Malawi, Ethiopia and Mozambique increased flexibility for MMD among PBFW using several strategies, including initiating all pregnant women on a three‐month supply of ART (3MMD) at the first ANC visit and initiating breastfeeding women on 3MMD between three and six months after delivery (P. Preko, CQUIN, ICAP and A. Wate, USAID/Mozambique personal communications). For pregnant women living with HIV who will not be able to return to the facility for delivery, providers should consider “mother‐baby packs” containing multi‐month ART for mothers, infant prophylaxis and cotrimoxazole with dosing instructions and a clear reminder of when and where EID testing can be completed. In some countries, HEI are now eligible for 3MMD with cotrimoxazole preventive therapy. Clinic staff and community cadres, especially OVC case workers, can provide instructions on these bundled services and offer virtual adherence follow‐up at intervals recommended in national guidelines [6].

For C/ALHIV, healthcare providers should maximize use of 3MMD for CLHIV two to five years of age, and six‐month dispensing (6MMD) of ART for C/ALHIV ≥ 5 years of age [28]. Given current manufacturing constraints of pediatric lopinavir/ritonavir solid formulations [29], this may result in providing 3MMD to all CLHIV ≥ 2 years of age and who weigh <20 kg, while providing 6MMD for C/ALHIV ≥ 20 kg. While some national guidelines may recommend that C/ALHIV, especially those initiating ART, return to the health facility sooner, it is unlikely that an interval dose change will be required during this time [17]. Virtual adherence and retention follow‐up for C/ALHIV and their caregivers that aligns with national guidelines appointment intervals will also reduce exposure risk and facility burden.

Special considerations should be given when including certain C/ALHIV in MMD. Caregivers of infants living with HIV should receive enough ART to last until the infant’s next routine immunization appointment. C/ALHIV who are doing well on second‐ or third‐line ART should receive at least 3MMD, even if such children do not qualify for MMD under normal circumstances [6]. C/ALHIV who are virologically unsuppressed should receive at least three months of their current regimen while working on adherence through monthly virtual assessment with facility, community and OVC staff.

Due to disruptions in global and national supply chains related to COVID‐19, countries should immediately implement inclusive MMD and promptly order ART regimens needed through the end of 2021, combined with supply chain modifications including reducing buffer stock and pushing ARVs out of central stores and into health districts, sites and patients’ hands [5, 6, 30].

### 2.4 Adaptations for C/ALHIV unstable on ART, including advanced disease, treatment failure and second‐/third‐line ART

C/ALHIV unstable on ART suffer from increased all‐cause morbidity and mortality [31]. For these C/ALHIV, it is important to quickly address failing ART regimens while prioritizing clinical support and virtual case management (including OVC services) when resources and staffing are limited. All children ≥20kg should be switched to a regimen with dolutegravir 50mg, and those failing non‐nucleoside reverse transcriptase inhibitor‐based therapy should be transitioned to a protease inhibitor‐based (if < 20kg) or dolutegravir‐based (if ≥ 20kg) regimen [32]. C/ALHIV who change regimens should receive at least 3MMD and should have virtual assessments at two weeks and then as needed if doing well. Moreover, viral load (VL) monitoring should not be a prerequisite to ART transitions.

C/ALHIV unstable on ART are a particularly vulnerable group that should be prioritized for clinical support and virtual case management when resources and staffing are limited. Every effort should be made to ensure families with unstable C/ALHIV can communicate with case managers and access OVC services [6]. Healthcare workers should provide caregivers with clear instructions and scenarios regarding when to seek clinical care via virtual means or in person. Caregivers living with HIV also need to be assessed to determine if they require additional support, as treatment failure in children is often linked to treatment failure in their caregivers [33].

### 2.5 Adaptations for VL monitoring for PBFW, infants, and C/ALHIV

Adapting VL monitoring frequency has the potential to preserve laboratory and transportation resources. Some countries have already suspended routine VL monitoring except for priority populations, including PBFW and C/ALHIV, and are relaxing VL monitoring prerequisites for MMD (e.g. Kenya, Malawi and Mozambique: P Preko, CQUIN, ICAP, personal communication). VL monitoring should be considered essential for PBFW, infants, and C/ALHIV and delivered with other clinical services in alignment with country guidance, especially for clients at risk of treatment disruption or with previously documented treatment failure [6]. Supporting clinical and laboratory staff with personal protective equipment and hand hygiene measures to ensure continuation of VL testing, especially for priority populations, should also be employed.

## 3 CONCLUSIONS

It is an immense global health challenge to protect families from acquiring SARS‐CoV‐2 while sustaining essential HIV services in the context of the COVID‐19 pandemic. Countries must take decisive action with enabling policies to ensure that targeted, family‐friendly programme adaptations are implemented for PBFW, infants, and C/ALHIV to limit SARS‐CoV‐2 transmission, while ensuring the continuation of high‐quality, life‐saving HIV services. It will be crucial for programmes to support programme adaptations using positive messaging to instil confidence in clients who may otherwise be fearful of accessing routine services due to COVID‐19 infection risk. The efficiencies and cost savings gained through these adaptations have the potential topreserve investments made in controlling the HIV epidemic and to protect clients and healthcare workers from COVID‐19, while also driving innovations that could result in more efficient and effective HIV programmes for years to come.

## COMPETING INTERESTS

None of the authors have competing interests to declare.

## AUTHORS’ CONTRIBUTIONS

The concept for this commentary was developed by AV, RG, MS, BRP, SS and MG. All authors contributed to the first draft. AV, RG, MS, NF, WPK, SS and MG revised the manuscript. MS coordinated review and approval from all authors for the final version.

## ABBREVIATIONS

CDC, Centers for Disease Control; CQUIN, The HIV Coverage, Quality, and Impact Network; ICAP, International Center for AIDS Care and Treatment Programs.
